# Characterizing oogenesis and programmed cell death in the eastern tree hole mosquito *Aedes (Protomacleaya) triseriatus*

**DOI:** 10.3389/finsc.2022.1073308

**Published:** 2023-01-16

**Authors:** Paul M. Airs, Michael J. Nazarchyk, Bradley J. Tucker, Lyric C. Bartholomay

**Affiliations:** ^1^ Department of Entomology, Iowa State University, Ames, IA, United States; ^2^ Department of Pathobiological Sciences, School of Veterinary Medicine, University of Wisconsin-Madison, Madison, WI, United States; ^3^ Midwest Center of Excellence for Vector-Borne Disease, University of Wisconsin-Madison, Madison, WI, United States

**Keywords:** nurse cell death, ovarian development, apoptosis, atresia, autophagy

## Abstract

Oogenesis in flies manifests as a carefully orchestrated cascade of developmental gates and growth events, punctuated by programmed cell death (PCD) and follicular resorption events. In anautogenous mosquitoes, a blood meal stimulates growth of primary follicles, but the timing of developmental stages is species-specific, and few species have been characterized. Here, we characterize the first gonotrophic cycle of oogenesis in *Aedes triseriatus* (Diptera: Culicidae), the principal vector of La Crosse Virus (LACV), a major cause of pediatric encephalitis in North America. We note significant differences in the timing and appearance of developmental stages from previous studies of other mosquito species, particularly *Aedes aegypti*. We also describe the appearance and timing of PCD events including atresia, nurse cell death, and follicular epithelium death and show that the majority of follicular epithelium cells do not undergo apoptosis during oogenesis but persist in the ovariole at least until the second gonotrophic cycle. This thorough characterization of oogenesis and PCD in *Ae. triseriatus*, through which LACV must persist in order to achieve filial infection, also serves as a baseline to study host-pathogen interactions during transovarial transmission.

## 1 Introduction

*Aedes* (*Protomacleaya*) *triseriatus* (Say), the Eastern Tree Hole mosquito, is found throughout deciduous forests of the Eastern half of North America (1) and is the principal vector of La Crosse Virus (LACV) (2, 3), the agent of La Crosse Encephalitis (4, 5). LACV is transmitted vertically in *Ae. triseriatus* through transovarial transmission in overwintering eggs and persists transstadially from larvae through to adults in the spring (6–9). The impact of LACV infection on *Ae. triseriatus* fitness is not fully understood, but transovarially infected LACV+ individuals maintain full reproductive capacity (10) and are more readily inseminated (11); however, embryonic mortality is increased in LACV+ eggs in diapause (12). To study the interaction between LACV and egg development, we reasoned that we must first characterize oogenesis in *Ae. triseriatus* as well as profile key programmed cell death (PCD) events essential to development and survival of resulting embryos.

Mosquitoes (Diptera: Culicidae), like many higher insects (Dermaptera, Psocoptera, Phthiraptera, and most holometabolous orders), have polytrophic meroistic ovaries containing a basal germarium with ovariole tubes, each housing a string of follicles that develop distally from the germarium (13). Each follicle within an ovariole is composed of a follicular epithelial layer that surrounds the oocyte along with a number of nurse cells (trophocytes) which provide the oocyte with protein and mRNA through cytoplasmic bridges (13, 14).

Dipteran oogenesis is an orchestrated cascade of developmental gates and growth events stimulated by nutrition, regulated by hormones and neurohormones (13, 15). Development is marked by developmental gates and programmed cell death (PCD) events driven by autophagy and apoptosis resulting in death of the entire follicle (resorption or atresia) or parts of the follicle which are no longer needed as development progresses including nurse cells and follicular epithelial cells (16). In higher flies, such as *Drosophila melanogaster, virilis, pseudoobscura*, and *gibberosa* (17, 18) as well as *Bactrocera dorsalis* (19) and *Ceratitis capita* (the medfly) (20), 14 morphological stages of oogenesis are evident. In mosquitoes, the timing and appearance of oogenesis parallels that of higher Diptera, but in many species is triggered by blood feeding (21, 22). Of the 3614 valid currently known species of mosquito (23), comprehensive descriptions of oogenesis are available just for *Aedes aegypti, Anopheles gambiae* and *Culex quinquefasciatus* (24). Seminal descriptions of oogenesis by Clements and Boocock (1984) and Christophers (11) scaffold the progression of oogenesis in distinct phases (previtellogenic, initiation, trophic, and post-trophic) (24), which are further divided into stages of development (G and then I-V) based on oocyte and follicle appearance and morphology (24, 25)(detailed in **Supplementary Table 1**).

In addition to morphological identifiers, PCD events in the ovary are essential processes and hallmarks of oogenesis. At mid-oogenesis, follicles of fruit flies and medflies either proceed through oogenesis or undergo follicular atresia (also known as oosorption or follicular resorption) *via* autophagy and apoptosis (16, 26, 27). Atresia occurs as a function of nutritional limitation in *D. melanogaster*, *Ae. aegypti*, and *Culex pipiens pallens* (16, 28–30). In *Ae. aegypti*, follicles that fail to develop at the same rate as surrounding follicles begin to show signs of atresia manifesting as bright crimson staining of follicular epithelial cells with neutral red (24). In higher flies, atresia occurs primarily through autophagy and apoptosis which manifest in condensation of nurse cell nuclei and later follicular epithelia cells as well as large vacuoles in the oocyte (27, 31). This mechanism may be preserved in mosquitoes, based on work done by Uchida et al. (2004) who reported hallmarks of apoptosis in epithelial cells of atretic follicles in *Culex pipiens pallens* (32).

In mid- to late-oogenesis, nurse cells undergo nurse cell death (NCD) to make room for the growing oocyte. In *D. melanogaster*, the nurse cells and oocyte form a syncytium connected by ring canal cytoplasmic bridges (33). These bridges facilitate transport of bulk cytoplasmic from the nurse cells to support growth and development of the oocyte. Once the oocyte reaches a threshold size and stage of development nurse cells dump remaining cellular contents to the oocyte and undergo NCD through autophagy and apoptosis (17, 34, 35). The interplay between autophagy and apoptosis during atresia and NCD has not been explored in detail in mosquitoes.

In late-oogenesis, sloughing of the follicular epithelium takes place leaving behind a chorion layer which becomes the egg shell (13). For the follicular epithelium to be removed in higher flies, death occurs in follicular epithelial cells during late oogenesis (36), however whether this occurs across all epithelia cells and the role of apoptosis in follicular epithelial removal in mosquitoes has not been demonstrated.

To better understand the role of PCD within the ovary during oogenesis in mosquitoes, and to provide the first description of oogenesis in *Ae. triseriatus*, we used PCD markers and morphological metrics to characterize follicular development and degradation during the first gonotrophic cycle. Here we provide an outline of the timing and traits of oogenesis observed in *Ae. triseriatus* following a bloodmeal for the first gonotrophic cycle as well as the fate of tissues and secondary follicles after oviposition. We demonstrate that both morphological staging and PCD events are highly synchronized processes both within and between individuals in *Ae. triseriatus*, and set the stage for future studies of the molecular underpinnings of PCD in mosquito oogenesis.

## 2 Materials and methods

### 2.1 Mosquito strains and rearing procedures

*Ae. triseriatus* larvae were reared from dried egg papers in enamel pans and fed daily with a slurry of ground TetraMin™ (Blacksburg, VA). Groups of 50 female pupae were collected ~24 hours prior to emergence and maintained post-eclosion on a 10% sucrose diet. Mosquitoes that received a bloodmeal were sucrose-starved for 18-24 hours prior to blood feeding, and provided with sucrose immediately after blood feeding until dissection. All life stages were maintained at 28 °C at 70% relative humidity with a 16:8 hour (light:dark) photoperiod.

### 2.2 Blood feeding

Adult female *Ae. triseriatus* were provided defibrinated sheep blood (HemoStat Laboratories, CA) through a Parafilm M^®^ (Bemis, Neenah, WI) membrane using a blown glass membrane feeder and were allowed to feed *ad libitum* (37). Following blood-feeding, unfed or partially fed individuals were removed from the study by aspiration. Observations were made with mosquitoes at three days or five days post eclosion (referred to as 3-days old and 5-days old in the text) at the time of blood feeding. The age of mosquitoes in the text references age at the time of bloodfeeding.

### 2.3 Dissections

Mosquitoes were dissected at specific time intervals as measured in hours post bloodmeal (hpbm) from 0-120 hpbm. At each timepoint, selected individuals were cold anesthetized at 4°C and immobilized on ice on filter paper in a glass Petri dish. Individual mosquitoes were dissected by inserting a minuten pin probe through the lateral thorax, ventral side facing upwards. Using forceps individuals were decapitated and then ovaries were removed from the 6^th^ and 7^th^ abdominal segments. Ovarioles containing primary follicles were separated from ovaries using probes for staining and imaging (for an illustration of an ovariole see **Figure S1**).

### 2.4 Neutral red staining and morphological measurements of healthy and atretic follicles

Dissected follicles from at least 10 individual adult females per timepoint were submerged in filtered 0.5% w/v neutral red (NR) in PBS for 10-30 seconds and rinsed in PBS 3 times immediately prior to bright field visualization. In each morphological analysis individual fully intact follicles were counted and measured according to visual cues previously described (see **Supplemental Table 1**) (24, 25). The number of follicles counted per timepoint per analysis were: area (n = 30), length and width (n = 50), proportional area of oocyte, nurse cell compartment, and follicular epithelium (n = 15). For quantification of atretic vs normally developing follicles, a total of 227-1098 follicles were counted per timepoint across 3 biological replicates; all unobscured follicles were counted and atresia was defined as the presence of punctate crimson red staining in the follicular epithelia (24, 28). More counts were made at some earlier timepoints due to eggs being smaller and more readily counted as well as egg batch sizes being larger prior to resorption of atretic follicles.

### 2.5 Acridine orange live cell staining and nurse cell death quantification

Dissected follicles from at least 10 individual adult females per timepoint were subject to analysis. Acridine orange staining was adapted from Abrams et al. (38) whereby dissected ovaries were rotated for 3 minutes in the dark in a 1:1 volume of acridine orange (10 µg/ml) in phosphate buffered saline and n-heptane for. Follicles then were washed 3 times in PBS and mounted in Fluoro-gel (EMS, Hatfield PA). Fluorescence was visualized immediately using a Nikon Eclipse 50i fluorescence microscope and NIS Elements D (Nikon, Melville, NY) with a Nikon B-2A long pass filter. As a control, one ovary of every pair was stained with NR. *Ae. triseriatus* follicles stained with acridine orange were measured from 3 or more biological replicates. Proportion of follicles with NCD were calculated based on the presence of ≥1 nurse cell with visible stain per follicle from acridine orange (n=191-231 per timepoint) and NR (n=227-710 per timepoint) stained follicles.

### 2.6 Fixation and immunofluorescence imaging

Follicles were fixed in 4% paraformaldehyde (in 0.1 M sodium phosphate buffer pH 7.4) for 30 minutes, washed twice in PBS, permeabilized (0.3% Triton X-100, 1% BSA, and 1% Sodium citrate in PBS), and washed twice more in PBS before staining. TUNEL staining (TMR Red *In Situ* Cell Death Detection Kit, Roche, Indianapolis, IN) was used to visualize late-stage apoptosis. Ovaries were transferred to the TUNEL reaction mixture for 2 hours at 37°C then rinsed 3 times in PBS. Positive controls were incubated in DNase I solution (0.1% BSA and 6u/ml DNase I in 50 mM Tris-HCl buffer) for 20 minutes at room temperature prior to staining. Negative controls were incubated in the absence of the enzyme terminal transferase. Follicles were co-stained with DAPI (25 μg/ml, Anaspec, CA) and Alexa Fluor 488 Phalloidin (0.835 μM, Life, NY) in PBS for 1 hour at room temperature. Processed ovaries were mounted in Vectashield (Vector Laboratories, CA) and visualized using a Nikon Eclipse 50i fluorescence microscope and NIS Elements D (Nikon, Melville, NY) with Nikon TRITC HYQ (TUNEL), B-2A (phalloidin) and UV-2E/C (DAPI) filters.

### 2.7 Imaging & data analyses

Photoshop CC (Adobe, San Jose, CA) photomerge tool was used to generate image composites to represent all follicles from each time-point within a single field of view. Image J (NIH, Bethesda, MD) was used to measure follicle length, follicle width, area of oocyte, nurse cell, and follicular epithelia, quantification of follicular atresia, and quantification of NCD. For measurements regarding size and shape, the polygon tool was used. The follicle perimeter was measured to calculate total area, the interior edge of the follicular epithelium was measured to calculate the interior follicle area (i.e., the oocyte & nurse cells), and the oocyte portion was measured using NR staining. Length and width measurements were made using the line tool measured at the longest and widest points of the follicle respectively. Follicle counts for quantifications were made using the multi-point tool. Measurements were plotted with Prism 6 (GraphPad Software, San Diego California USA).

## 3 Results

### 3.1 A timeline of oogenesis in *Ae triseriatus*

To define the timing of oogenesis in *Ae. triseriatus*, 3-day old virgin females fed with defibrinated sheep blood were assessed using frameworks for oogenesis from previous descriptions (24, 25). Morphological characteristics including the appearance of the oocyte nucleus, oocyte content as a proportion of the total follicle, the follicle length to width ratio, and the total follicle area were considered as defining features of different phases and stages of development (**Table 1**; **Table S1**). In addition, PCD events including follicular atresia and NCD were critical to marking Trophic phase IIIb and IVb respectively (See **Table 1**; **Table S1**). Finer observations of these PCD and morphological events are described below.

**Table 1:** Timing of ovarian developmental stages in *Ae. triseriatus*.

Phase	Stage	Oocyte*	Key event	Ref Figure	HBPM
**Previtellogenic**	G-Ia		Oocyte not visible	**Figure S1**	PreBM
	Ib – IIb	0-10%	Oocyte nucleus visible	**Figure S1**	0-8
**Initiation**	IIIa	≤ 50%	NR in oocyte	**Figures S1E**, **1A**	08-24
**Trophic**	IIIb	50-75%	Peak atresia	**Figure 3**	18-36
	IVa	~ 90%	Size and oocyte content increase	**Figures 2A, C**	36-60
	IVb	90-100%	Nurse cell death	**Figure 4**	48-72
			Narrowing follicle	**Figure 2B**	
**Post-Trophic**	V	100%	Follicle at maximum length	**Figure 2B**	72-96
			follicular epithelium removal	**Figure 1H**	

* Percentage of follicle interior area as compared to nurse cell area.

### 3.2 Morphometrics of ovarian development

**Figure 1:**
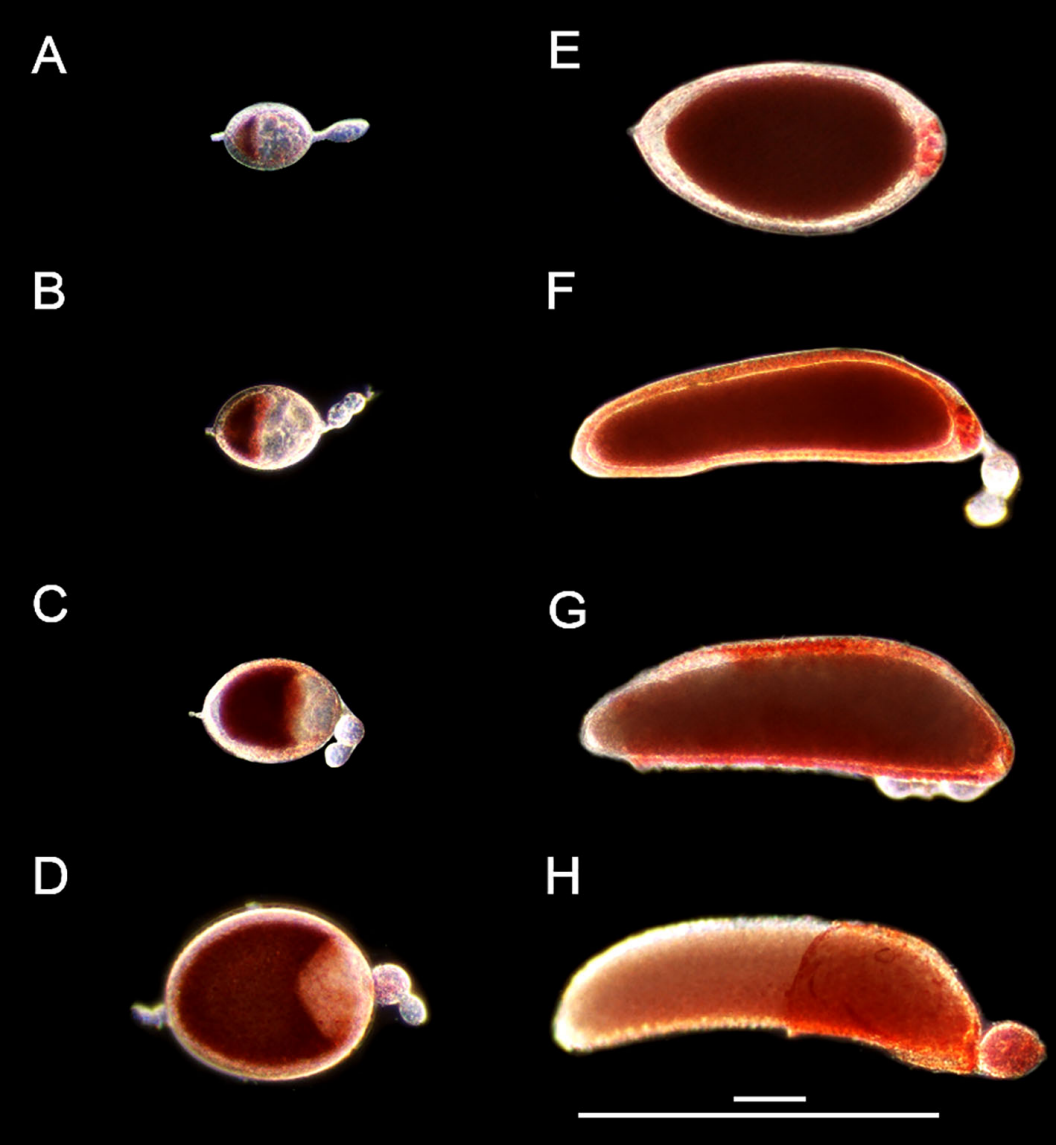
Follicle morphology post bloodmeal. Live NR stained primary follicles from 3-day old *Ae. triseriatus* during **(A)** early initiation phase IIIa at 8 hpbm, **(B)** late initiation phase IIIa at 12 hpbm, **(C)** trophic phase IIIb at 24 hpbm, **(D)** trophic phase IVa at 46 hpbm, **(E)** trophic phase IVb at 55 hpbm and **(F)** 60 hpbm, **(G)** post trophic phase V at 72 hpbm and **(H)** 96 hpbm. Scale bars = 100 µM and 500 µM. Secondary and tertiary follicles within the ovariole visible in all parts except **(E)** For an illustration of follicle morphology please see **Figure S1**.

**Figure 2:**
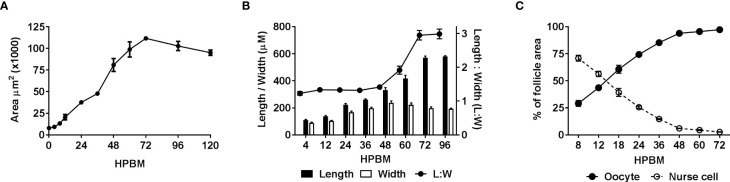
Quantifying changes in follicle morphology. The size and shape of follicles including the oocyte and nurse cell compartments were measured over time following a bloodmeal. **(A)** Follicular area over time n = 30, **(B)** follicular length, width and ratio (Length : Width) over time n = 50, **(C)** comparison of oocyte and nurse cell area as a percentage of inner follicle area over time n = 15. Data are the average of 3 or more biological replicates (± SEM).

The first gonotrophic cycle in *Ae. triseriatus* was measured from 0 to 120 hpbm using neutral red (NR) to mark the shape and size of primary follicles (**Figures 1**, **2**). Primary follicles are the posterior-most follicles in each ovariole of the ovary and develop following ingestion of a bloodmeal in anautogenous mosquitoes. Follicles can be divided into three main cell types, the oocyte (developing egg), nurse cells, and a follicular epithelial layer (illustrated in **Figure S1**).

Immediately following a bloodmeal oocyte nuclei are visible (**Figure S1**). In 3-day old mosquitoes the oocyte cytoplasm is typically not stained with NR at 4 or 8 hpbm (**Figures S1A, B**; **1A**), but uptake of NR does appear in some follicles as early as 4 hpbm increasing to 100% of follicles by 12 hpbm (**Figure S1E**). From 0-12 hpbm follicles double in size and by 24 hpbm follicles grow 12-14 times compared to pre-bloodmeal (**Figure 2A**). Follicles remain relatively spherical with no major change until 48 hpbm, whereupon follicles narrow and lengthen while continuing to gain mass (**Figure 2B**). The oocyte itself is relatively indistinguishable from 0-8 hpbm but increases in size compared to the nurse cells until 60 hpbm, at which time the oocyte occupies almost the entirety of the follicle interior (**Figures 1A–F**, **2C**). However, the follicular epithelium also grows proportionally to the entire follicle occupying ~25% of the area from 8-72 hpbm (**Figure S3**). From 72-96 hpbm, the epithelium sloughs off the follicle leaving behind a characteristic chorion structure (**Figure 1H**). During the latter stages of oogenesis, secondary follicles also grow, reaching the size of initiation phase II follicles by 96 hpbm (**Table S1**, compare **Figures 1H**; **S1A**).

Beyond the first gonotrophic cycle, secondary follicles develop beyond the pre-vitellogenic phase preceding a second blood meal, possibly utilizing nutrition recuperated from resorbed follicles or follicular content such as the follicular epithelial layer from primary follicles or nurse cell remnants (expanded on below), or leftover from the initial bloodmeal. Pre-oviposition, with primary follicles in stage V still in the ovariole, secondary follicles are in previtellogenic stages Ia-IIa and tertiary follicles remain at stage G (**Figure S4A**). Near-immediately post-oviposition, intact primary follicle follicular epithelium is evident, and secondary follicles contain a visible follicular epithelium and oocyte nuclear envelope marking them at stage IIb (**Figure S4B**). Twenty hours post-oviposition, the primary follicle follicular epithelium is no longer visible, and secondary follicles appear at stage IIIa marked by NR staining in the oocyte, and the oocyte containing <50% of the total follicle area but with the oocyte nucleus occluded (**Figure S4C**).

### 3.3 PCD during oogenesis

#### 3.3.1 Follicular atresia

**Figure 3:**
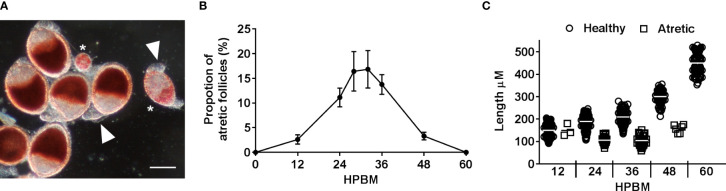
Characterizing follicular atresia. **(A)** Representative NR stained healthy follicles, atretic follicles (asterisks), and unstained secondary follicles (arrowheads) at 24 hpbm. Scale bar = 100 µM. **(B)** The proportion of atretic follicles per ovary over time post bloodmeal. **(C)** Length of healthy vs atretic follicles over time. Data are the average of 3 replicates (± SEM).

Atretic follicles were identified using several visual cues including bright red NR-stained vesicles within the follicular epithelium, asynchronous and smaller and/or rounder follicles, and a loss of clear follicular cell types (**Figure 3A**). Atresia was observed from 12-48 hpbm and peaks with ~17% of follicles undergoing resorption between 24-36 hpbm (**Figure 3B**). All atretic follicles observed were equivalent or smaller in length to the average 12 hpbm follicle (**Figure 3C**).

**Figure 4:**
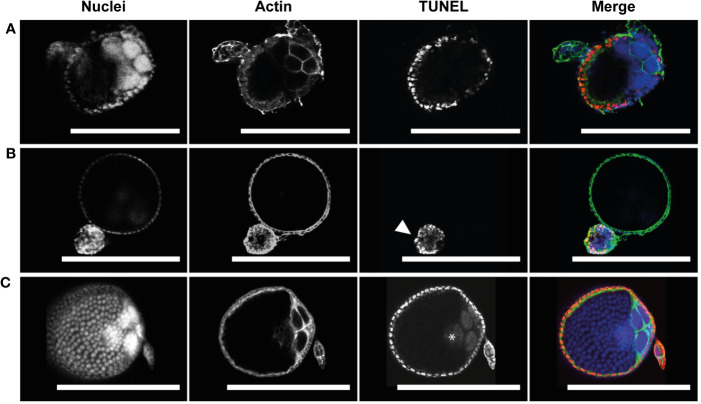
Follicular atresia is apoptotic in *Ae. triseriatus*. **(A)** Early-stage atresia in a primary follicle with intact DAPI positive nurse cell nuclei and apoptotic TUNEL positive follicular epithelial cells. **(B)** An atretic and normally developing primary follicle. Advanced atresia with TUNEL positive follicular epithelia is evident in the atretic primary follicle (arrowhead); note no clear definition between follicular epithelium, oocyte, and nurse cells. As compared to a cross section of a normally developing primary follicle above. **(C)** Positive control primary and secondary follicles treated with DNase I showing TUNEL positive nuclei in follicular epithelial cells, nurse cells, and the oocyte (asterisk). Nuclei (DAPI), actin (Phalloidin), and TUNEL staining channels shown. Scale bar = 100 µM. For an illustration of follicle morphology please see **Figure S1**.

To assess whether PCD was occurring in atretic follicles, TUNEL staining of was used to reveal evidence of DNA fragmentation. TUNEL staining evident in follicular epithelial cells of atretic follicles (**Figure 4**). Neither the oocyte or nurse cell nuclei were TUNEL positive in early atretic follicles (**Figure 4A**) or more advanced atretic follicles (**Figure 4B**). This result emulates that of NR stained atretic follicles (compare **Figures 3A**, **4B**).

#### 3.3.2 Nurse cell death

**Figure 5:**
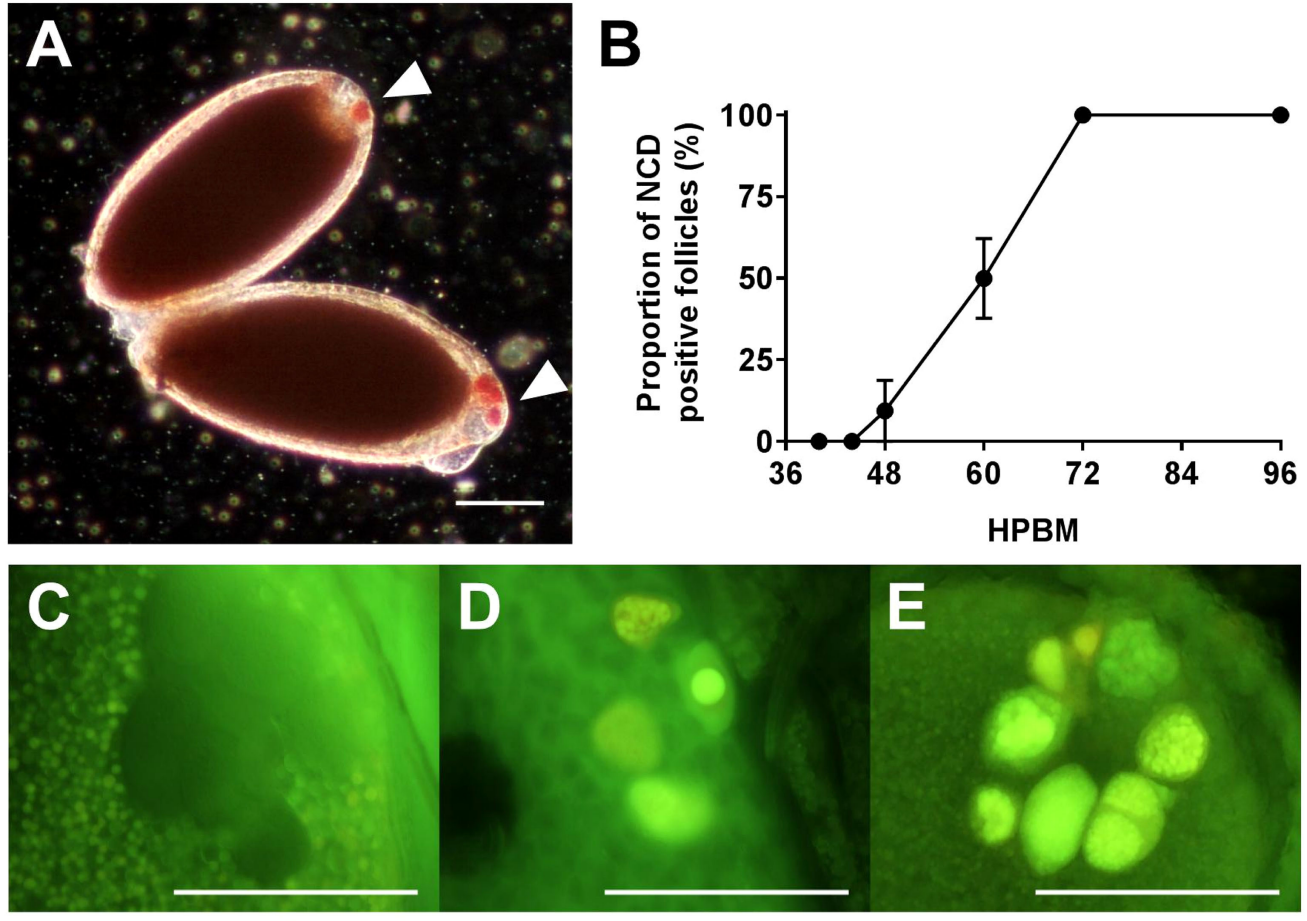
Timing Nurse Cell Death (NCD). **(A)** NR stained NCD positive follicles at 60 hpbm with 1 or more dying nurse cells (arrowheads). **(B)** Proportion of NCD positive follicles per ovary over time. **(C–E)** Acridine orange stained nurse cells at 58 hpbm displaying **(C)** impermeable nurse cells pre-NCD, **(D)** NCD positive nurse cells with intact nuclei, and **(E)** NCD positive nurse cells with condensed and degraded nuclei. Scale bars = 100 µM. Data are the average of 3 or more biological replicates (± SEM).

The progression of NCD was measured by NR and acridine orange staining (**Figures 5**; **S5**). NCD first appears with permeabilization of individual nurse cell nuclear envelopes at 46 hpbm (**Figure 5A**) with 50% of follicles undergoing NCD by 60 hpbm in 3-day old adults (**Figures 5B**; **S5A**). In older mosquitoes (5-days old at the time of bloodfeeding) follicles undergo NCD earlier with 50% undergoing NCD by 52 hpbm (**Figure S5B**). By 72 hpbm, nurse cells are no longer distinguishable. During the process of NCD, nurse cells condense and stain bright crimson with NR as a marker of acidification. This staining is first localized to the nurse cells (see **Figure 1E**), but nurse cell remnants are later visible on the follicular epithelium (**Figures 1F, G**).

**Figure 6:**
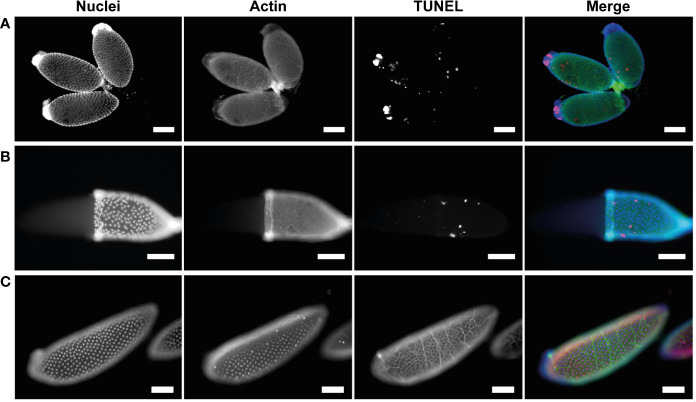
Apoptotic cell death in nurse cells and follicular epithelium is asynchronous. **(A)** Healthy primary follicles at 60 hpbm during mid-oogenesis displaying various stages of NCD and sporadic follicular epithelial death. **(B)** Healthy primary follicle during late-oogenesis as 96 hpbm undergoing follicular epithelial sloughing with majority of follicular epithelial nuclei intact. **(C)** DNase I positive TUNEL staining control of healthy primary follicle with follicular epithelia during late-oogenesis at 96 hpbm. Nuclei (DAPI), actin (Phalloidin), and TUNEL staining channels shown. Scale bar = 100 µM.

NCD is asynchronous both between and within follicles, with follicles displaying anywhere from 1-7 apoptotic nurse cells from 46-72 hpbm (**Figures 5C–E**). Over time, progressively more nurse cells undergo NCD and become increasingly compartmentalized as contents are lost to the oocyte or phagocytosed by the follicular epithelium (**Figures 1E–G**, **5D, E**). The compaction of nurse cells is coupled with binucleation and a shift of green to red staining and nuclear fragmentation indicating acidification of cellular microvesicles (stained by acridine orange) or release of RNA from the nucleus (**Figures 5C–E**). Compaction, fragmentation of nuclei, and staining with acridine orange indicates apoptotic type death (16, 38). To confirm apoptosis type DNA fragmentation, a sign of late apoptosis, TUNEL staining was performed at 60 hpbm on follicles with normal morphology and revealed a portion of TUNEL positive nurse cells in 2/3 follicles displayed (**Figure 6A**). TUNEL, NR, and acridine orange staining were in agreement in that NCD occurs asynchronously.

#### 3.3.3 Follicular epithelium death

To determine whether cells of the follicular epithelium undergo apoptosis in order to be removed TUNEL staining was performed at 60 hpbm and 96 hpbm during mid- and late-oogenesis respectively (**Figures 6A, B**). Apoptotic DNA fragmentation was noted in a small number of scattered cells in both mid- and late-stage follicles, but the majority of nuclei appear normal and do not stain with TUNEL compared to the positive control (**Figure 6C**). In these cells actin is also noticeably uncoordinated. Whether actin dysregulation is a function of apoptosis or caused by mechanical damage to individual cells was not determined. To detect whether the epithelium undergoes apoptosis prior to or post oviposition, TUNEL staining was performed on segmented ovaries at 120 hpbm but no cells appeared TUNEL positive at this time either pre-, during-, or post-oviposition (**Figures S6A–C**). Removal of the follicular epithelium is thought to be driven by apoptosis prior to removal from the egg at the anterior pole after deposition of chorion in higher flies (13, 39). By contrast, while some cells undergo apoptosis asynchronously in *Ae. triseriatus* (**Figures 6A, B**), the entire epithelium containing many intact cells is dislodged from the follicle prior to oviposition in what appears to be a coordinated mechanism in *Ae. triseriatus* (see **Figure 1H**). Follicular epithelial cells are apparent in cases after oviposition between 120-144 hpbm (**Figure S4B**) but are not visible in others where secondary follicles are developing (**Figure S4C**) and so may undergo cell death or become phagocytosed following oviposition.

## 4 Discussion

### 4.1 Characterizing stages of oogenesis in *Ae. triseriatus* by morphometrics and PCD

In this study, 3-day-old lab-reared *Ae. triseriatus* were exposed to defibrinated sheep blood and fed until repletion; the follicles in this scenario reach maturity (stage V) by 96 hpbm. It should also be noted that in *Ae. triseriatus* the type of blood given, the age, and the sexual naivety of the female impact the timing of oogenesis and egg batch (40, 41). Under the same conditions, *Ae. aegypti* (Liverpool) had developmental staging comparable to previous descriptions using human or guinea pig blood (24) and reached stage V maturity as soon as 72 hpbm. By comparison, *Ae. aegypti* and *Ae. caspius* take 60-68 hpbm and 56 hpbm to reach stage V, respectively (24, 42).

#### 4.1.1 The previtellogenic phase: Stages G, Ib, IIa, IIb

Oogenesis events in an anautogenous mosquito precede the bloodmeal; in the previtellogenic phase (stage G), follicular progenitor cells replicate in the germarium then are separated in ovarioles (43). Primary follicles are the distal-most follicles of each germarium. These develop a follicular epithelium layer, and the oocyte becomes distinguishable from the nurse cells (Stage Ia-Ib). Next, the oocyte becomes more recognizable with the formation of lipid droplets, while the oocyte nucleus and nucleolus remain visible by reflected light (stage IIa-IIb). Throughout the previtellogenic phase, follicles are resistant to NR staining. We find that immediately following a bloodmeal, *Ae. triseriatus* follicles are in previtellogenic stages Ib-IIb. Herein, follicles appear clear and spheroid with oocyte nuclei visible clearly at 150x magnification and no positive staining with NR in the oocyte (**Figure S1A**). By 4 hpbm, most follicles remain at previtellogenic stages Ib-IIb and appear largely unchanged in size and shape (**Figures S1B**; **2A**).

#### 4.1.2 The initiation phase: Stage IIIa

In anautogenous oogenesis, ingestion of a bloodmeal stimulates release of ovary ecdysteroidogenic hormone (OEH) and insulin-like peptides (ILPs) from neurosecretory cells in the brain that stimulate ecdysteroid hormone (ECD) production in the ovaries (22, 44–47). At the same time, blood cholesterol is absorbed into follicle epithelial cells is converted to ecdysone that stimulates vitellogenin production in the fat body (15). The release of OEH and ILPs, and interaction with other pathways such as the Target of Rapamycin (TOR) also regulate midgut gene expression to digest the bloodmeal and initiate yolk protein production in the fat body and uptake of yolk proteins in the oocyte in lipid vesicles (15, 22, 48, 49). OEH, ILPs and ECD therefore act as the gate between the previtellogenic phase and the initiation phase (**Table S1**), which is marked by NR staining of lipid vesicles in oocytes (**Figure 1A**). In 3-day old *Ae. triseriatus*, no follicles stain positive with NR at 0 hpbm, indicating that no follicles pass the initiation gate before or immediately following a bloodmeal, and that follicles require time for yolk protein production and uptake from the fat body (**Figure S1E**). This is supported by the presence of clearly visible oocyte nuclei, even at 4 hpbm, characteristic of Stage IIb and Christophers’ Stage 2 (**Table S1**; **Figures S1A, B**). Occlusion of the oocyte nucleus, another mark of stage IIIa, occurs between 4 - 12 hpbm as yolk protein uptake continues and 100% of follicles stain positive with NR (**Figures 1A, B**; **S1E**). In older individuals (5 day post-eclosion AKA 5-day old adults) follicles were larger at 0 hpbm and stained positive with NR at 0 and 4 hpbm (**Figures S1C, D**), indicating that some vitellogenesis and receptivity to yolk protein uptake occurs prior to or immediately following a bloodmeal. The oocyte nuclei of 5-day-old adults were still visible in many follicles, despite positive NR staining (**Figures S1C, D**). Therefore, it may be that NR staining is not strictly indicative of stage IIIa in *Ae. triseriatus* if oocyte nuclei are still visible as this is a hallmark of phase IIb (**Table S1**). Ovaries from older adults (up to 11-days) were assessed but were indistinguishable from 5-day old adults.

#### 4.1.3 The trophic phase: Stages IIIb, IVa and IVb

Oocyte content was tracked as a percentage of overall follicular area as a key indicator of trophic phases (**Figure 1C**). At 12 hpbm, visible oocyte content reaches 43% of the follicle, increasing to 60% by 18 hpbm (**Figure 2C**). Because the cutoff for stage IIIa is defined as 50% oocyte content, stage IIIb must begin between 12 and 18 hpbm. Stage IIIb marks the start of the trophic phase, which is not defined by a change in shape, but an increase in oocyte content from 50-75% alongside an increase in follicle size. Another marker of stage IIIb in *Ae. aegypti* is peak follicular atresia, which may in this case act as a secondary stage marker (24). Here we quantified atretic follicles and discovered a distinct peak from 24-36 hpbm (**Figure 3B**). Based on size increase, lack of shape change, and peak atresia, stage IIIb occurs in *Ae. triseriatus* at 18-36 hpbm.

At the point of transition to trophic stage IVa, *Ae. triseriatus* deviates from the criteria set forth previously for other mosquitoes. Stage IVa is characterized by continued growth, change in shape of the follicle and thinning of the follicular epithelium, up to 90% oocyte content, and intact nurse cells (see **Supplemental Table 1**). In *Ae. triseratus*, the criteria for trophic stage IVa for oocyte content are met at 18-24 hpbm, but follicle size continues to increase without a shape change until 48-60 hpbm (**Figures 1A, B**). The morphological criteria also fit using this timeframe because follicle growth continues up beyond 60 hpbm (**Figure 2A**) and follicular epithelium thinning starts between 48-60 hpbm (**Figure 2B**). We observed that stage IVa occurs in the window of follicular growth between two major cell death events: atresia (during stage IIIb) and NCD (during stage IVb). Since peak atresia ends at 36 hpbm (**Figure 3B**) and NCD does not occur in the majority of follicles until 60 hpbm (**Figure 5B**), stage IVa occurs between 36 and 60 hpbm for the first gonotrophic cycle.

The end of the trophic phase marks the transition to stage IVb. At this point, the oocyte encompasses almost 100% of the follicular interior and the follicle assumes the shape of the mature egg. These criteria set stage IVb between 48-72 hpbm (compare **Figures 1E–G**, **2A, B**). Similar to late oogenesis in *Drosophila*, nurse cells undergo NCD when the oocyte nears maturity (36, 50). NCD is a hallmark of stage IVb and is evidenced by bright crimson staining of nurse cells using NR (**Figures 1E, F**, **5A**). This event is first observed at 48 hpbm in *Ae. triseriatus*, but does not peak until 60 hpbm (**Figure 5B**). Finally, Clements & Boocock note that in stage IVb, the “follicle assumes the shape of the mature oocyte, or almost so, but has not reached full length” (24). In *Ae. triseriatus*, follicles reach maximum length at 72 hpbm.

#### 4.1.4 The post-trophic phase: Stage V

The post-trophic stage marks the final events of oogenesis that occur in rapid succession. Here follicles reach maximum length, shed the follicular epithelia, and chorionic structures become visible. In this study, at 72 hpbm, 100% of nurse cells were degraded indicating the end of the trophic phase (**Figure 5B**). Follicles at this time also reach a size maximum and thinning halts (**Figures 2A, B**). By 96 hpbm, chorionic structures are also visible and the follicular epithelium detaches, revealing the fully developed oocyte (**Figures 1F, G**). We consistently observed the follicular epithelium rolling onto itself and down the length of the follicle while still in the ovariole.

#### 4.1.5 Secondary follicle development initiates prior to a second bloodmeal in *Ae. triseriatus*

During the first gonotrophic cycle, secondary follicles grow and mature from stage G to initiation phase II by 96 hpbm (compare **Figures 1H**; **S1A**). Prior to oviposition, secondary follicles do not pass into the initiation phase but stain with NR throughout (**Figure S1A**). Following oviposition, the primary follicle follicular epithelium remains in the ovary and is resorbed over ~24 hours (**Figures S1B, C**). Resorption of the follicular epithelium appears to be essential to clear the distalmost region the ovariole to allow secondary follicles to assume this position (**Figure S1B**). Immediately following oviposition, the follicular epithelium remains intact and secondary follicles do not pass into stage IIIa, but show characteristics of stage IIb (**Figure S1B**; **Table S1**). Resorption of the follicular epithelium is associated with the secondary follicles entering stage IIIa; as such, the follicular epithelium may provide nutrition sufficient for development of some secondary follicles without need for a second bloodmeal (**Figure S1C**).

### 4.2 Programmed cell death during oogenesis

This study focused solely on PCD events during undisrupted oogenesis within the ovary. It is key to note that PCD events outside of the ovaries are critical for oogenesis to occur and are discussed extensively elsewhere (15), such as autophagy of the fat body for regulation of vitellogenin production during egg maturation (15, 51, 52).

#### 4.2.1 Follicular atresia

In insects, atresia occurs during early-mid stages of oogenesis (28). The process necessitates termination of yolk deposition in the oocyte and complete degradation of the follicle. In other fly species (e.g., *D. oleae* and *D. melanogaster*) atresia occurs as a function of PCD in the form of apoptosis and/or autophagy (26–28, 53). In *Ae. aegypti*, atresia is noted during stage IIIa and peaks during stage IIIb between 25-30 hpbm (24). Our results show atretic follicles as early as 12 hpbm, which aligns with stage IIIa morphological data (**Figure 3B**). All of the atretic follicles observed in *Ae. triseriatus* were equivalent or smaller in length to the average 12 hpbm follicle (**Figure 3C**), which may indicate that a nutritional deficiency/sufficiency gate occurs at this time point or follicle size. Although atresia has not been used to define this stage in *Ae. aegypti*, it is informative in *Ae. triseriatus*. This may be especially useful for defining stage IIIb in *Ae. triseriatus* because the transition from IIIa to IIIb in this mosquito does not fit the criteria for other species (**Table S1**, section 3.3.3).

Interestingly atretic follicle oocytes and nurse cell nuclei did not stain with TUNEL, but the follicular epithelium did. This result emulates that of NR stained atretic follicles (compare **Figures 3A**, **4B**). Atresia may be localized or controlled by the follicular epithelium in *Ae. triseriatus*; this is congruent with atresia in *Culex pipiens pallens* wherein active caspases are restricted to the epithelial cells of atretic follicles (32). Similar results were seen in *Plasmodium*-infected *Anopheles stephensi*, wherein atretic follicles exhibited apoptosis mainly in the follicular epithelium (54). By contrast, in the higher flies, *D. oleae, D. melanogaster*, and *Ceratitis capitate*, apoptosis was evident in the nurse cell compartment of atretic follicles (20, 27, 36, 39).

#### 4.2.2 Nurse cell death

Nurse cells are fundamental to oogenesis in many multicellular organisms and function in much the same way in flies as in *Caenorhabditis elegans* and *Hydra* (50). In *Aedes* species, each follicle contains a single oocyte and 7 nurse cells that provide cytoplasmic material including mRNA and protein directly to the oocyte *via* ring canals (43). After the oocyte has sufficiently grown and developed, the nurse cells degrade and die. In *Drosophila*, NCD starts with permeabilization of the nuclear membrane, followed by transportation or ‘dumping’ of cytoplasmic contents (including large amounts of RNA and protein) to the oocyte through the ring canals, and finally degeneration and apoptosis of the remaining cell (50, 55). To decipher the similarities of NCD between *Drosophila* and *Ae. triseriatus*, numerous staining techniques were employed. In *D. melanogaster*, acridine orange staining produces green/red nuclei during NCD, particularly in nuclei that are permeabilized as a function of an apoptotic event (38, 54). We witness the same permeabilization in nuclei which occurs sporadically between nuclei within and between follicles with anywhere from 1-7 nurse cells undergoing NCD at any given time from 46-72 hpbm (**Figure 5B**). Nurse cells decrease in size, and shift from green to red staining with acridine orange (**Figures 5D, E**). The decrease in nurse cell size is possibly due to cytoplasmic dumping, while the shift in color is either due to acidification of the cell during apoptosis, or increase in RNA production based on the staining characteristics of acridine orange (56). Additionally, red-stained vesicles were often seen dispersing from the nurse cell compartment, suggesting that acridine orange may mark RNA or acidic vesicle movement from the nurse cell compartment to the oocyte. Nurse cells undergoing NCD also displayed increased DNA fragmentation in TUNEL assays and as such are likely undergoing apoptosis (**Figure 6A**). Acidification of microvesicles within the nurse cells is likely driven by autophagy, which would explain the shift from green to red staining in nurse cells undergoing NCD. However, further studies are required to confirm whether autophagy drives NCD along with apoptosis in *Ae. triseriatus* in the same way as observed in higher flies (35).

#### 4.2.3 Follicular epithelial death

The epithelium is a dynamic and multifunctional cell layer and constitutes a significant area (between 15 and 30%) of the overall follicle size (**Figure S3**). Ultimately, the epithelium deposits a protective vitelline chorion layer (43), and at the post trophic phase (stage V), the epithelium is sloughed to release the mature oocyte (**Figure 1H**). This phenomenon is different from *Drosophila* and other higher flies where follicular epithelial cells undergo apoptosis while attached to the developing follicle, starting from the anterior pole, are sloughed away from the follicle, and then engulfed by epithelia of the oviduct (36, 57). To investigate whether these cells undergo apoptosis while still associated with the follicle, TUNEL staining was performed (**Figure 4**; **Figure S6**). The results show that only a small number of epithelial cells stain with TUNEL, with the remaining displaying large, fully formed nuclei typical of healthy cells, but are sloughed away from the developed stage V oocyte prior to oviposition. The removal of viable follicular epithelial cells in *Ae. triseriatus* indicates that there is more research to be done to reveal the mechanism behind epithelium death and cell sloughing. Additionally, TUNEL positive follicular epithelial cells could reflect phagocytosed remnants of degraded nurse cells, as is seen in *D. melanogaster* (39). After removal from developed primary follicles, follicular epithelial cells stained crimson with NR, but this staining may be remnant material from NCD and not a result of acidification or chromatin condensation (see **Figure S4**). Positive control TUNEL stain was observed in primary follicle epithelium pre- and post- sloughing as well as secondary follicle and the ovarian sheath epithelia (**Figure S6**). Therefore, epithelial cells do not appear to be resorbed during the first gonotrophic cycle and may be retained for longer periods in *Ae. triseriatus*. Interestingly, at 120-144 hpbm secondary follicles were observed developing past the previtellogenic phase into stage IIIa with no observable remnants of the primary follicle epithelium (see **Figures S4B, C**) and so it is likely that nutrients from the follicular epithelia are utilized to initiate vitellogenesis production and growth of secondary follicles without a secondary bloodmeal. However, whether the oviduct absorbs the follicular epithelium, and the timing and appearance of follicular epithelial cell death remains to be discovered in *Ae. triseriarus*.

## 5 Conclusions

Herein we provide a comprehensive description of PCD events and morphological changes during oogenesis in *Ae. triseriatus* (see **Table 1**). The criteria used to morphologically characterize the phases of oogenesis in *Ae. aegypti* and other mosquito species do not fully capture the nature of some key stages and phases of oogenesis in *Ae. triseriatus*. Our analysis of PCD events in *Ae. triseriatus* oogenesis aligns with reports from higher Diptera, including the presence of apoptotic follicular epithelial nuclei in atretic follicles as well as apoptosis and probable autophagy driving nurse cell death. However, novel mosquito-specific and potentially species-specific phenomena occur in mid- and late-stage oogenesis. For example, the death of follicular epithelial cells but not oocyte or nurse cells as a hallmark of atresia in *Ae. triseriatus* is in accordance with findings in *Culex pipiens* (32) and is distinct from higher-flies (27, 31). These finding add weight to the follicular epithelial layer having a primary role in dictating atresia in mosquitoes. Late in oogenesis, PCD was not observed in association with the sloughing of follicular epithelial cells from the fully-formed follicle. Together, these results provide a characterization of oogenesis in this key vector of La Crosse virus and for the first time delineate the timing of PCD events as part of normal ovarian development in anautogenous mosquitoes.

## Data availability statement

The raw data supporting the conclusions of this article will be made available by the authors, without undue reservation.

## Author contributions

PA was responsible for: Data Curation, Formal Analysis, Investigation, Methodology, Visualization, Writing – Original Draft Preparation, Writing – Review and Editing. MN was responsible for: Investigation. BT was responsible for: Data Curation, Formal Analysis, Investigation, Methodology. LB was responsible for: Conceptualization, Funding acquisition, Investigation, Methodology, Project administration, Supervision, Writing – original draft, Writing – review and editing. All authors contributed to the article and approved the submitted version.
