# Supplementary material for: Characterizing oogenesis and programmed cell death in the eastern tree hole mosquito Aedes (Protomacleaya) triseriatus
Source: Front Insect Sci. 2023 Jan 16;2:1073308. doi: 10.3389/finsc.2022.1073308 (PMC10926484; doi:10.3389/finsc.2022.1073308)
Supplement: Supplementary file 1 [file DataSheet_1.docx]

Supplementary Figures


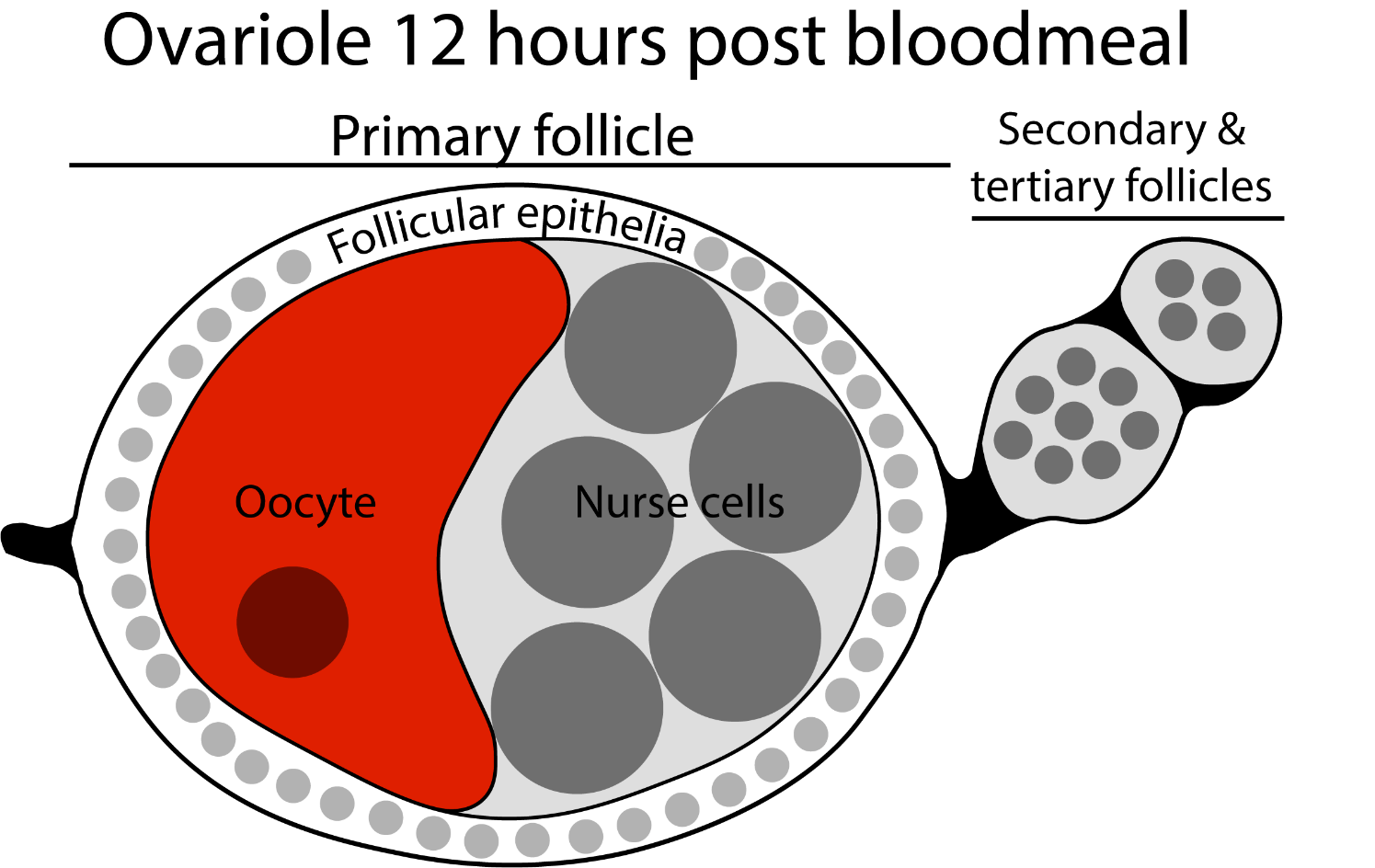


**Supplementary Figure 1. Illustration of an *Ae. triseriatus* ovariole 12 hours post bloodmeal.** Illustration includes 5/7 nurse cells as some are typically occluded based on the field of view, the oocyte is shown in red as is presented following neutral red staining. The oocyte nucleus is shown but is usually occluded following uptake of vitellogenin. Secondary and tertiary follicles are shown and do not have distinct cell types under live cell imaging. The oviduct is shown in black.


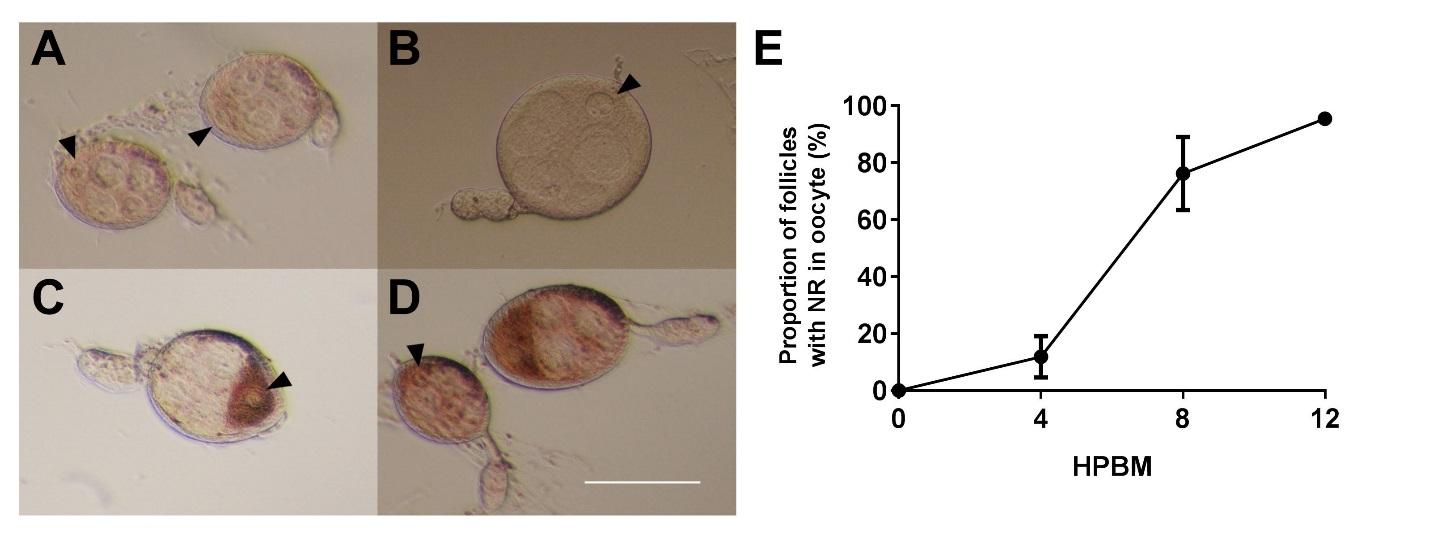


**Supplementary Figure 2. Follicles switch from previtellogenic to initiation phase based on mosquito age.** Follicles from 3-day old adult mosquitoes at (A) 0 hpbm and (B) 4 hpbm and follicles from 5-day old adult mosquitoes at (C) 0 hpbm and (D) 4 hpbm. (E) Proportion of follicles from 3-day old adult mosquitoes with NR staining in the oocyte over time (*n* = 79-467). Arrowheads indicate oocyte nuclei. Scale bar = 100 µM. All data are the average of 3 or more biological replicates (± SEM), *n* = number of follicles measured (from 10-15 mosquitoes) per time point.


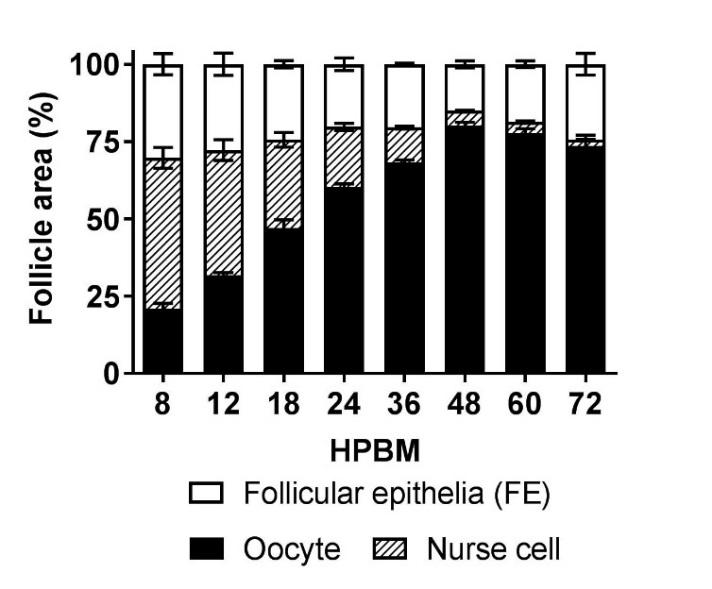


**Supplementary Figure 3. Follicular Epithelial layer grows proportionally with the follicle during oogenesis.** Proportion of follicle area encompassed by the follicular epithelium, oocyte, and nurse cell compartment following a bloodmeal. The average of 3 or more biological replicates (± SEM).


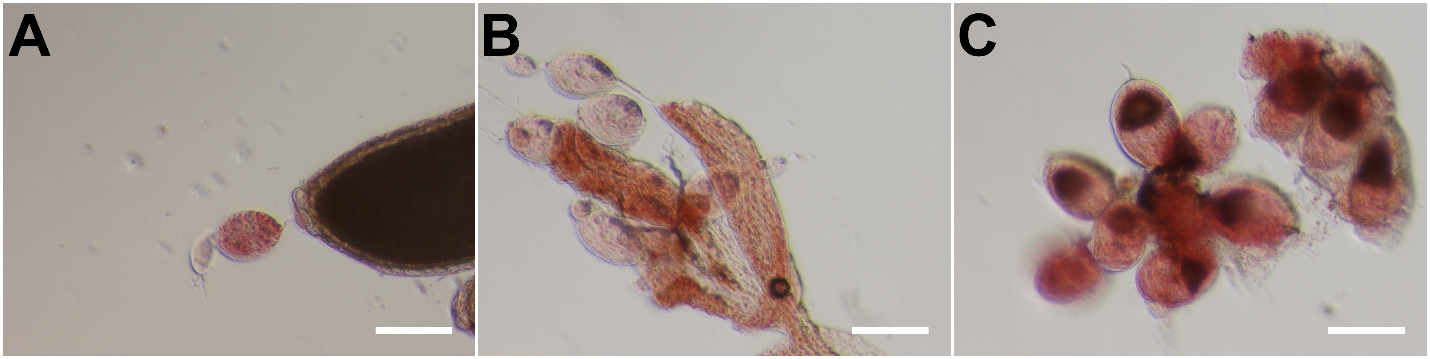


**Supplementary Figure 4. Initiation phase IIIa reached by secondary follicles following oviposition without a second bloodmeal.** Follicles at 120-144 hpbm pre- and post- oviposition. (A) 120 hpbm pre-oviposition with primary follicle with attached follicular epithelium at stage V, secondary follicle at stage Ia-IIa with unusual staining, and tertiary follicle at stage G. (B) 144 hpbm post-oviposition, with intact primary follicle follicular epithelium, secondary follicles with visible follicular epithelium, and oocyte nuclear envelope at stage IIb, and tertiary follicles at stage G. (C) 144 hpbm post-oviposition, with mostly resorbed primary follicle follicular epithelium and secondary follicles at stage IIIa. Scale bar = 100 µM.


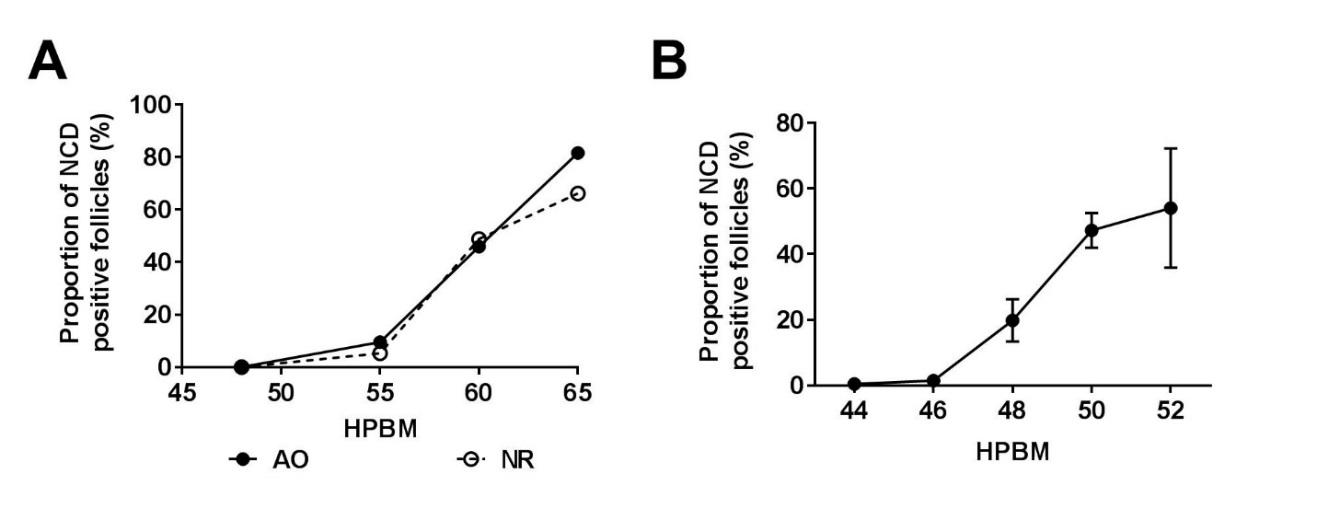


**Supplementary Figure 5. Additional studies of NCD.** (A) Quantification of NCD compared between acridine orange and NR staining methods over time. (B) Proportion of NCD positive follicles per ovary in 5-day old adult mosquitoes using acridine orange. All data are the average of 3 or more biological replicates (± SEM in part B).


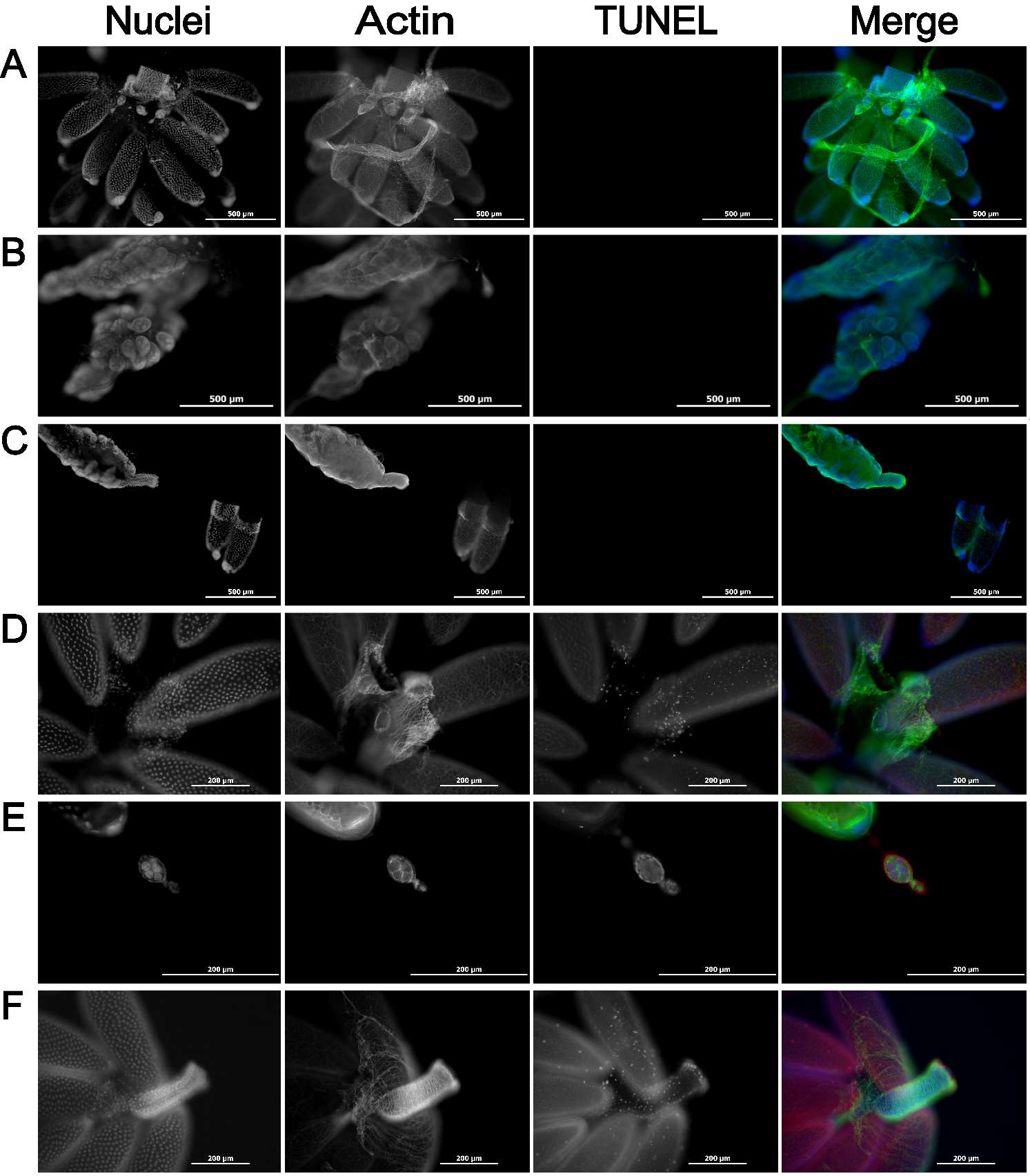


**Supplementary Figure 6. Apoptosis does not occur during late-stage oogenesis.** Ovaries imaged at 120 hpbm including nuclei (DAPI), actin (Phalloidin), and TUNEL staining. (A-C) Primary follicles, secondary follicles, and ovarian sheath showing no TUNEL staining in ovaries from individuals in conditions (A) pre-oviposition, (B) post-oviposition, and (C) during incomplete oviposition where some developed eggs remain. Incomplete oviposition is common in *Ae. triseriatus* where skip-oviposition occurs allowing multiple oviposition sites per egg batch. (D-F) DNase I positive control primary follicles, secondary follicles and ovarian sheath pre-oviposition.
