# Supplementary material for: Characterizing oogenesis and programmed cell death in the eastern tree hole mosquito Aedes (Protomacleaya) triseriatus
Source: Front Insect Sci. 2023 Jan 16;2:1073308. doi: 10.3389/finsc.2022.1073308 (PMC10926484; doi:10.3389/finsc.2022.1073308)
Supplement: Supplementary file 2 [file Table_1.docx]

Supplementary Tables

**Supplementary table 1. Criteria for developmental staging of follicles during the ovarian cycle.**

| Author: | Clements & Boocock | | | | Christophers | | |
| --- | --- | --- | --- | --- | --- | --- | --- |
| Species: | *Aedes aegpyti, Culex pipiens, Anopheles gambiae** | | | | Anophelines | | |
|  | **Phase** | **Stage** | **Description** | **Developmental gate (stimulus)** | | **Stage** | |
| Definitions | Previtellogenic | G | Follicles partially separated from germarium. | Germinal (20-Hydroxyecdysone) | | Not described | |
|  |  |  | Oocyte not entirely surrounded by follicular epithelium. |  |  |  |  |
|  |  | Ia | Follicle separate from germarium, |  | | Stage 1: oocyte free from lipid granules | |
|  |  |  | Oocyte entirely surrounded by follicular epithelium but not distinct from nurse cells. |  |  |  |  |
|  |  | Ib | Oocyte distinguishable from nurse cells. | Stage I  (Juvenile hormone) | |  |  |
|  |  |  | No visibility of lipid at 200X magnification. |  |  |  |  |
|  |  | IIa | Lipid visible at 200X magnification. | Pre-vitellogenic (unknown) | | Stage 2: yolk granules present, but not obscuring nucleus | |
|  |  | IIb | Lipid visible at 20-50X magnification. |  | |  |  |
|  |  |  | No inclusions stained by neutral red. |  |  |  |  |
|  |  |  | Oocyte nuclear membrane & nucleolus visible. |  |  |  |  |
|  | Initiation | IIIa | Ooplasm clouded with inclusions visible at 10X magnification. | Stage III  (ovary ecdysteroidogenic hormone (OEH) and insulin-like peptides (ILPs) stimulate ecdysteroid hormone (ECD)) | | Stage 3: oocyte nucleus obscured, follicle still oval shape. | |
|  |  |  | Large yolk spheres visible. |  |  |  |  |
|  |  |  | Several follicles lag and are resorbed. |  |  |  |  |
|  |  |  | Oocyte clouded by yolk and progressively occupies up to 50% of the follicle length. |  |  |  |  |
|  | Trophic | IIIb | Follicle increases in size but no change in shape. |  |  |  |  |
|  |  |  | Oocyte occupies up to 75% of follicle length. |  |  |  |  |
|  |  |  | Many follicles degenerate at this stage. |  |  |  |  |
|  |  | IVa | Follicle grown further and shape change begins as follicles narrow. |  | | Stage 4: follicle elongate and shape of mature egg | |
|  |  |  | Nurse cells remain intact. |  |  |  |  |
|  |  |  | Oocyte occupies up to 90% of follicle length. |  |  |  |  |
|  |  |  | From this stage on most follicles reach maturity. |  |  |  |  |
|  |  | IVb | Follicle assumes shape of mature oocyte but not full length. |  | |  |  |
|  |  |  | Nurse cells degenerate & stain bright crimson. |  |  |  |  |
|  |  |  | Chorionic structures appear. |  |  |  |  |
|  | Post-trophic | V | Oocyte at full length, | Maturation (Syngamy ) | | Stage 5: chorionic structure visible | |
|  |  |  | Follicular epithelium degenerates and, |  |  |  |  |
|  |  |  | Chorionic structures become fully formed. |  |  |  |  |
| Citations | (Clements, 1992; Clements and Boocock, 1984) | | | | (Christophers, 1911) | | |
| * *Anopheles* have no clear stage Ib and oocyte growth is 50% of the follicle length by IIa. | | | | | | |  |
